# A CD Study of a Structure-Based Selection of *N*-Heterocyclic Bis-Carbene Gold(I) Complexes as Potential Ligands of the G-Quadruplex-Forming Human Telomeric hTel23 Sequence

**DOI:** 10.3390/molecules29225446

**Published:** 2024-11-19

**Authors:** Maria Marzano, Filippo Prencipe, Pietro Delre, Giuseppe Felice Mangiatordi, Gabriele Travagliante, Luisa Ronga, Gennaro Piccialli, Michele Saviano, Stefano D’Errico, Diego Tesauro, Giorgia Oliviero

**Affiliations:** 1 Centro di Servizio di Ateneo per le Scienze e Tecnologie per la Vita (CESTEV), University of Napoli Federico II, Via Tommaso De Amicis 95, 80145 Napoli, Italy; maria.marzano@unina.it; 2 Department of Chemical and Pharmaceutical Sciences, University of Trieste, Via Licio Giorgieri 1, 34127 Trieste, Italy; filippo.prencipe@units.it; 3 Institute of Crystallography (IC), CNR, Via Amendola 122/O, 70126 Bari, Italy; pietro.delre@unina.it (P.D.); giuseppefelice.mangiatordi@cnr.it (G.F.M.); 4 Department of Pharmacy, University of Naples Federico II, Via Domenico Montesano 49, 80131 Napoli, Italy; picciall@unina.it; 5 Department of Chemical Sciences, University of Catania, Viale Andrea Doria 6, 95125 Catania, Italy; travagliantegab@gmail.com; 6 Institute of Analytical and Physical Chemistry for the Environment and Materials (IPREM-UMR 5254), Université de Pau Et Des Pays de L’Adour, E2S UPPA, CNRS, 64053 Pau, France; luisa.ronga@univ-pau.fr; 7 Institute of Crystallography (IC), CNR, Via Vivaldi 43, 81100 Caserta, Italy; michele.saviano@cnr.it; 8 Interuniversity Research Centre on Bioactive Peptides (CIRPEB), University of Naples Federico II, 80134 Naples, Italy; 9 Department of Molecular Medicine and Medical Biotechnology, University of Napoli Federico II, Via Sergio Pansini 5, 80131 Napoli, Italy; golivier@unina.it

**Keywords:** auranofin, cancer, chemotherapy, *N*-heterocyclic carbene gold(I) complexes, G-quadruplex, CD spectroscopy, molecular docking, hTel23, telomeres, telomerases

## Abstract

Herein, we report the structure-based selection via molecular docking of four *N*-heterocyclic bis-carbene gold(I) complexes, whose potential as ligands for the hTel23 G-quadruplex structure has been investigated using circular dichroism (CD) spectroscopy, CD melting, and polyacrylamide gel electrophoresis (PAGE). The complex containing a bis(1,2,3,4,6,7,8,9-octahydro-11*H*-11*λ*^3^-pyridazino[1,2-a]indazol-11-yl) scaffold induces a transition from the hybrid (3 + 1) topology to a prevalent parallel G-quadruplex conformation, whereas the complex featuring a bis(2-(2-acetamidoethyl)-3*λ*^3^-imidazo[1,5-a]pyridin-3(2*H*)-yl) moiety disrupted the original G-quadruplex structure. These results deserve particular attention in light of the recent findings on the pathological involvements of G-quadruplexes in neurodegenerative diseases.

## 1. Introduction

**Figure 1:**
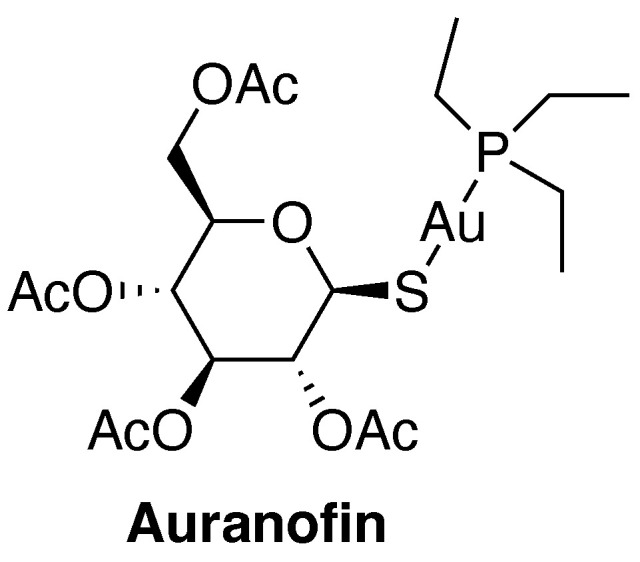
The structure of Auranofin.

The fight against cancer needs many different weapons, and inorganic medicinal agents may play a significant role in this issue. Platinum-containing drugs are widely employed as chemotherapeutic agents against testicular, ovarian, and breast cancers [1,2,3]. However, a prolonged cisplatin treatment may lead to drug resistance [4] and often induces severe side effects (e.g., nephrotoxicity, neurotoxicity, ototoxicity) [5]. To overcome these serious drawbacks, other transition metals have been explored for the construction of complexes endowed with anticancer properties [6,7]. Among them, gold complexes have gained much attention because they act in innovative ways with respect to the classical platinum complexes [8]. Currently, 2,3,4,6-tetra-*O*-acetyl-l-thio-β-d-glyco-pyranosato-*S*-(triethylphosphine)-gold(I)] (auranofin, Figure 1) is the unique gold drug utilized in clinical practice to treat rheumatoid arthritis; however, since 1985, it has been studied for its potential anticancer properties. How the cytotoxic activity of gold(I) complexes takes place is still a topic of wide discussion. Clear evidence is based on the poor reactivity with the DNA double helix and the ability to produce severe mitochondrial damage [9]. Most of these effects can be attributed to the strong and selective inhibition of the seleno-enzyme thioredoxin reductase (TrxR), which is involved in the maintenance of the intracellular redox balance [10]. Auranofin can act in alternative pathways inhibiting the proteasome, modulating specific kinases, or showing antitelomerase activity [11].

In the last decade, the fast development of *N*-heterocyclic mono-carbene and bis-carbene gold(I) (NHC–gold(I)) complexes as catalysts in many types of reactions have suggested their use in the fields of medicinal chemistry, especially as potential chemotherapeutic agents [12]. NHC ligands offer a suitable mode to stabilize the metal center, tuning the lipophilicity and the physiochemical properties of the complexes via the establishment of direct metal−carbon bonds. Within this context, the research on NHC–gold(I) complexes as potential antiproliferative agents has strongly increased [13]. Among the possible biological targets of NHC–gold(I) complexes [14] telomeres have been studied only in the last decade [15,16]. Telomeres consist of repetitive nucleotide sequences and associated proteins that protect the ends of chromosomes from deterioration or fusion with neighboring chromosomes. The very end of the telomere has a guanine-rich single-stranded 3′ overhang that can form G-quadruplex secondary DNA structures [17,18]. G-Quadruplexes are involved in several biological processes [19], such as telomere homeostasis [20], cancer progression [21], and neurodegenerative diseases [22]. During cell replication, telomeres shorten until the cell undergoes death. In certain tumors, the telomerase enzyme is overexpressed, preventing the telomeres from becoming shortened. Since the presence of G-quadruplexes has been demonstrated in vivo [23,24], a promising anticancer strategy is to evaluate the effect of small molecules on the formation of G-quadruplexes at the telomeric level [25]. To this aim, natural [26,27,28] and synthetic [29,30] compounds have been tested in vitro on model telomeric G-quadruplex-forming sequences [31]. Among the synthetic compounds, transition metal complexes have attracted much attention from medicinal chemistry [32]. In fact, the chemical diversity resulting from the coordination geometries of the ligands around the metal centers can strongly influence the binding mode of the complexes with the G-quadruplexes and affect the biological properties.

The discovery that NHC–gold(I) complexes can target the model human telomeric G-quadruplex-forming sequences [15,33] prompted researchers to tune the nature of the ligands around the metal with the aim of obtaining more effective and selective binders [34]. From these studies, it was possible to deduce that the presence of lipophilic NHC ligands around the positively charged gold(I) center could account for the interaction with the unusual DNA secondary structures [35].

**Figure 2:**
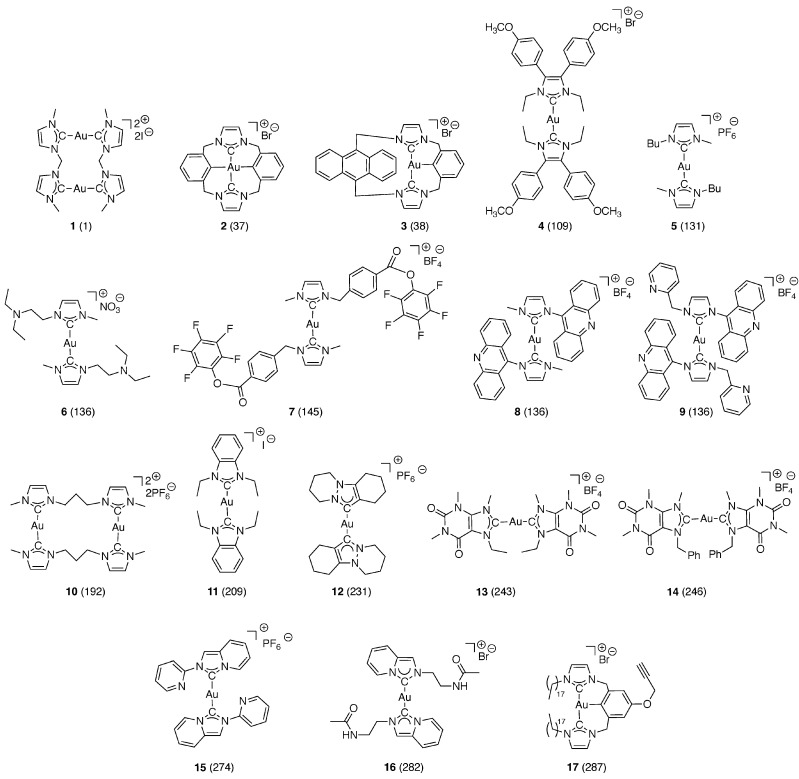
The structures of the NHC–gold(I) complexes **1**–**17** selected by the molecular docking simulations. The numbering is in parentheses according to Porchia et al. [13].

Recently, Porchia et al. [13] reported on a systematic classification of all the NHC–gold(I) complexes synthesized from 2004 to 2016 and rationalized the biological data based on their chemical structures. Seventeen NHC–gold(I) complexes, extracted from the collection (Figure 2), were found to interact with the crystallographic parallel hTel23 G-quadruplex sequence, as demonstrated by molecular docking simulations performed in this study. Since the hTel23 G-quadruplex predominantly adopts an intramolecular hybrid (3 + 1) structure in solution, we assessed the ability of the four most interesting NHC–gold(I) complexes **4**, **9**, **12**, and **16** to induce conformational changes in the G-quadruplex structure using circular dichroism (CD), CD melting, and polyacrylamide gel electrophoresis (PAGE) experiments. An optimized synthesis of complex **16** is also reported.

## 2. Results and Discussion

### 2.1. Molecular Docking Simulations

**Figure 3:**
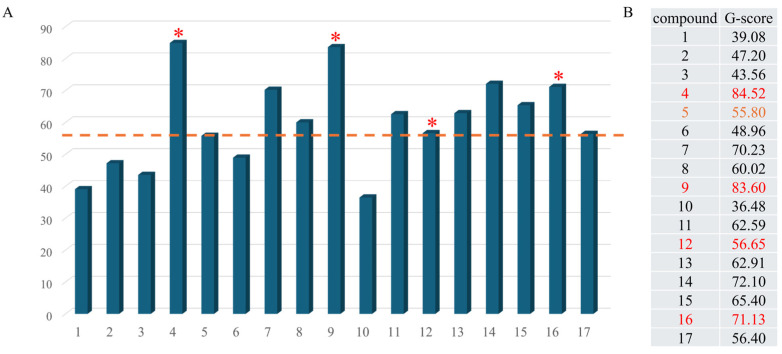
Panel (**A**): Chart showing the G-score (y-axis) calculated for each complex (x-axis). The orange dashed line represents the reference G-score of complex **5**. The red asterisks represent the selected complexes **4**, **9**, **12**, and **16**. Panel (**B**): Table summarizing the calculated G-scores for each complex. The complex marked in orange corresponds to complex **5**, while the complexes marked in red represent complexes **4**, **9**, **12**, and **16**.

As the first step of our work, we filtered the library of the NHC–gold(I) complexes described by Porchia et al. [13], based on synthetic accessibility, antiproliferative activity, and chemical class. Accordingly, we selected 17 compounds (Figure 2 and Figure 3) that showed interesting in vitro antiproliferative activities with IC_50_ values in the nmol/L or μmol/L range and for which the mechanism of action is not yet fully understood.

**Figure 4:**
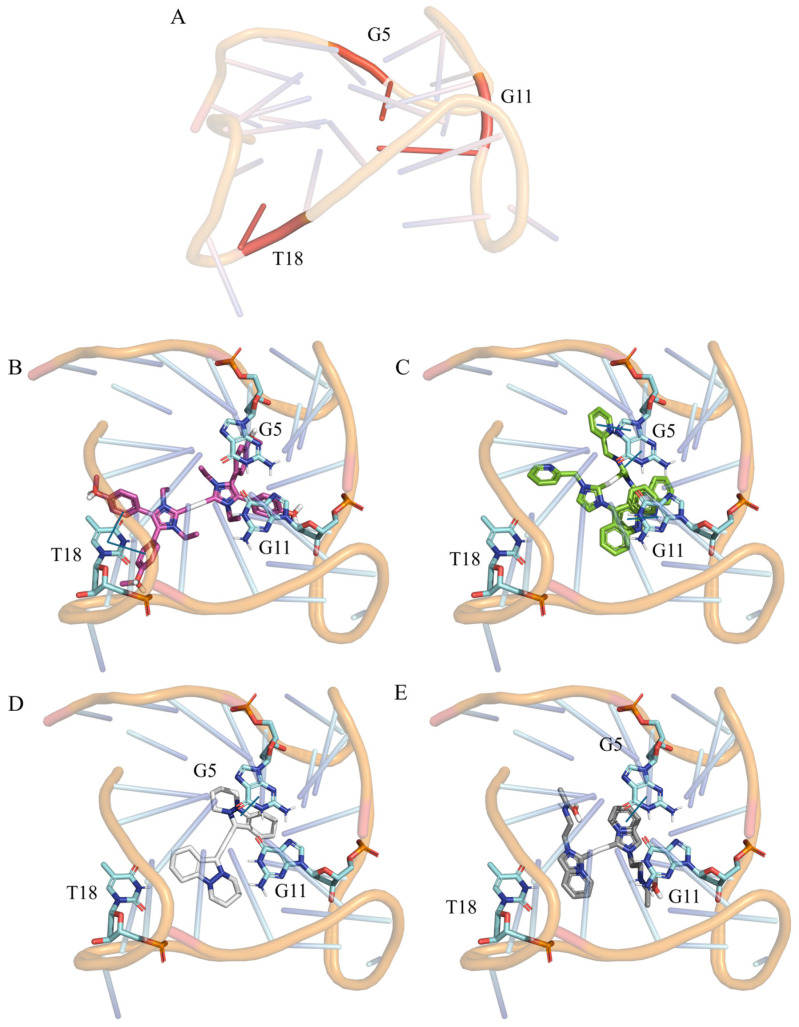
Panel (**A**): X-ray solved hTel23 G-quadruplex structure extracted from the Protein Data Bank (PDB code: 6H5R). Panels (**B**–**E**): Top-scoring docking poses within the hTel23 G-quadruplex (PDB: 6H5R) of complexes **4**, **9**, **12**, and **16**, respectively. For clarity, only polar hydrogen atoms are shown. Important residues are rendered as sticks, while the hTel23 G-quadruplex is represented as cartoon. π−π interactions are depicted as blue lines.

The present study aimed to investigate the ability of the selected complexes to interact with telomeric G-quadruplexes, which were revealed to be valuable targets of anticancer drugs. For this purpose, we used a crystal structure that represents the only known structure of a gold(I) dicarbene antitumor drug (**5**) in complex with the human telomeric hTel23 parallel G-quadruplex (pdb code: 6H5R) [33]. This crystal structure was particularly suitable for our study due to the high chemical similarity between the selected complexes and complex **5**, and its high resolution (2.0 Å). To address the limitations of traditional docking methods in simulating metal-containing complexes, we adopted the protocol developed on the GOLD platform by Sciortino et al. [36], which demonstrated high accuracy in predicting the binding poses for 39 high-quality metal compound-protein complexes (see Section 3 for details). The docking results, shown in Figure 4, reveal a distribution of G-scores, suggesting potential binding to the G-quadruplex for several of the investigated complexes. Building on the obtained results, we selected four complexes (i.e., 4, **9**, **12**, and **16**) displaying both G-scores and binding poses similar to complex **5** (Figure 4).

Complex **4** is a cationic bis[1,3-diethyl-4,5-diarylimidazol-2-ylidene]gold(I) complex with *para*-OCH_3_ substituents in the aromatic rings, displaying an IC_50_ of 0.17 ± 0.05 μmol/L against MCF-7 cell lines [37]. Docking simulations predicted for **4** a good computed G-score (64.52 kcal/mol), and a pose that established well-oriented π–π interactions with T18, G5, and G11 residues. Complex **9**, a gold(I)–acridine-based *N*-heterocyclic carbene, is particularly compelling due to the intrinsic fluorescent properties conferred by the acridine moiety and its high predicted G-score (83.60 kcal/mol), the highest among the selected complexes. Complex **9** interacts with the G-quadruplex via π–π contacts with G5 and G11 residues. This gold(I) complex exhibited specificity for the MiaPaca2 cancer cell line in the μmol/L range [38]. Complex **12**, with an IC_50_ of 2.35 ± 0.07 μmol/L against NCI-H1666 cells [39], interacted with G5 and returned a G-score of 56.65 kcal/mol. Finally, complex **16**, an in vivo suppressor of melanoma tumor growth via the modulation of p53 and other apoptotic factors (p21, NF-κB, VEGF, and MMP-9) [40], displayed a computed G-score of 71.13 kcal/mol and an interaction with G5. Our findings suggested that complexes **4**, **9**, **12**, and **16**, as well as complex **5**, could bind as planar structures through favorable π–π contacts with the hTel23 G-quadruplex, which is in full agreement with the experimental coordinates of complex **5**.

### 2.2. Synthesis of Complexes ***4***, ***9***, ***12*** and ***16***

**Scheme 1:**
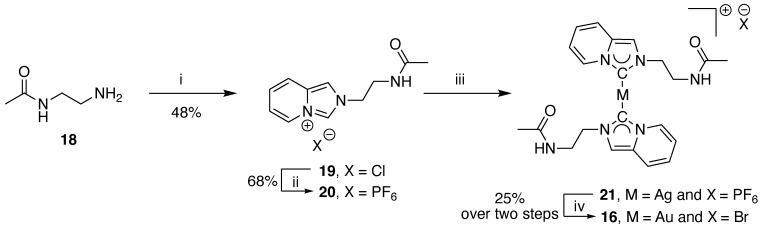
Reagents and conditions: (i) (CH_2_O)_n_, EtOH, r.t., 4 h, then 2-Pyridinecarboxaldehyde, HCl, EtOH, r.t., 16 h; (ii) KPF_6_, H_2_O, r.t., 1 h; (iii) Ag_2_O, 10% MeOH in CH_2_Cl_2_, r.t., 16 h; (iv) AuClS(CH_3_)_2_, LiBr, 10% MeOH in CH_2_Cl_2_, r.t., 5 h.

Complexes **4**, **9**, and **12** were synthesized following the reported procedures [41,42]. Conversely, the synthesis of complex **16** reported by Aron et al. [43] was optimized (Scheme 1). In the first step for the synthesis of complex **16**, the primary amine of compound **18** was reacted with paraformaldehyde, and then 2-pyridinecarboxaldehyde was added, affording the 2-(2-acetamidoethyl)-2*H*-imidazo[1,5-*a*]pyridin-4-ium **19** as Cl^-^ salt. After salt metathesis (**19**→**20**) and the formation of the Ag(I) complex **21** (not isolated) [44], a transmetallation reaction was performed in situ, yielding the bis-NHC–gold(I) complex **16** as a bromide salt. The structures of complexes **4**, **9**, **12**, and **16**, ascertained by NMR and ESI MS analyses, were consistent with data from the literature.

### 2.3. CD Titration Studies Among Complexes ***4***, ***9***, ***12***, and ***16*** and the Model Human Telomeric G-Quadruplex-Forming hTel23 Oligonucleotide

CD spectroscopy has become a highly useful tool for detecting the topology of G-quadruplex structures [45] and their changes upon interaction with ligands [46,47,48]. CD melting studies can be performed to gain insights into the evaluation of the stability of G-quadruplexes after the interaction with ligands [49].

**Figure 5:**
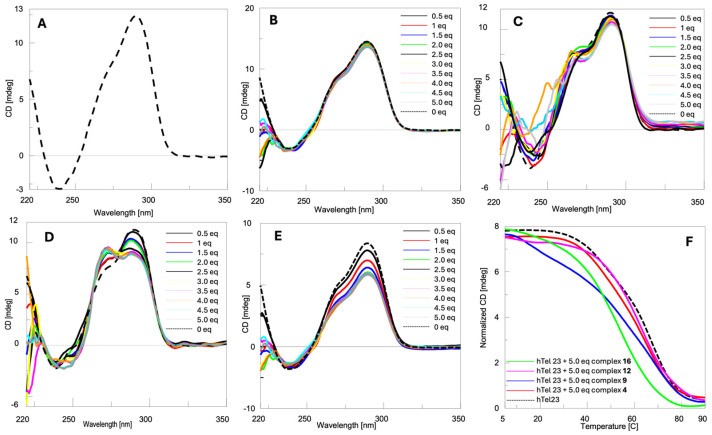
CD spectra of hTel23 G-quadruplex in the absence (**A**) and presence of increasing molar equivalents of complexes **4** (**B**), **9** (**C**), **12** (**D**), and **16** (**E**). CD melting spectra of hTel23 G-quadruplex in the absence and presence of 5 equiv. of complexes **4**, **9**, **12**, and **16** (**F**). CD spectra were acquired in a 90 mmol/L K^+^ phosphate-buffered solution (pH = 7.3). CD melting experiments were acquired by monitoring the CD value of the higher positive Cotton effect in the temperature range of 5–90 °C in a 90 mmol/L K^+^ phosphate-buffered solution (pH = 7.3).

The CD spectrum of the hTel23 oligonucleotide, annealed in a 90 mmol/L K^+^ phosphate-buffered solution (pH = 7.3), displayed the main species as an intramolecular hybrid (3 + 1) G-quadruplex structure with a positive band at 290 nm, a shoulder at 272 nm and a weak negative band at 240 nm (Figure 5A and Table 1), in agreement with data from the literature [33,50]. Additionally, the CD_290_ melting profile of the hTel23 G-quadruplex resulted in a sigmoidal curve with a melting temperature (T_M_) of 67 °C (Figure 5F and Table 1) [51].

Before performing the CD titration experiments, we evaluated the aggregation properties of the complexes **4**, **9**, **12**, and **16** in a 90 mmol/L K^+^ phosphate-buffered solution (pH = 7.3) by monitoring the increment of the absorbance at their λ_max_ upon the addition of increasing amounts of complexes (0.5–5 equivalents, steps of 0.5). For all the complexes reported in this study, aggregation phenomena could be excluded, as a linear increase in absorbance vs. concentration was obtained in all the ranges of concentrations explored (Figures S1–S4, Supplementary Information).

The CD titration spectra of complexes **4**, **9**, **12**, and **16** incubated with the hTel23 G-quadruplex are reported in Figure 5B–E, respectively. DMSO was used to dissolve complexes **4**, **9**, **12**, and **16**, obtaining a maximum concentration of the organic solvent (0.9%) that was not detrimental to the G-quadruplex structure [49].

When the hTel23 G-quadruplex was incubated with increasing amounts of complexes **4** or **9**, the original G-quadruplex profile was almost retained in both cases, along with the T_M_ values extracted from the respective CD_290_ melting curves. These results may indicate that for both complexes, the formation of external adducts did not alter the original G-quadruplex profile, as recently reported for similar compounds [33].

To unravel the mechanism of action of complex **9** against the MioPaca2 cancer cell line, Gimeno et al. [38] hypothesized the involvement of telomeric G-quadruplexes and their stabilization by virtue of the intercalative features of complex **9**. Our data allowed the discarding of such a behavior for complex **9**.

Upon the addition of increasing amounts of complex **12** to the hTel23 G-quadruplex, a transition from the hybrid (3 + 1) topology to a prevalent parallel G-quadruplex conformation occurs, as evidenced by the increased intensity of the peak at 272 nm, accompanied by the reduction in the intensity of the peak at 290 nm [52]. Similar behaviors were reported recently by Vilar et al. [53] and Paulo et al. [54] during their evaluation of the ligand properties of a series of metal–salphen/salen derivatives and (iso)quinolinyl-pyridine-2,6-dicarboxamides toward hTelo sequences, respectively. Adding increasing amounts of the non-metal ligand to the hTel23 G-quadruplex did not cause any changes to the CD spectrum, emphasizing the crucial role of the Au center in the transition from the hybrid (3 + 1) to the parallel conformation. The CD_290_ melting profile of the hTel23 G-quadruplex incubated with 5 equiv. of complex **12** gave a well-defined sigmoidal curve with a TM = 63 °C.

Ligands that can promote switches between G-quadruplex topologies are particularly interesting. In fact, either the stabilization/destabilization of a G-quadruplex structure or a conformation transition upon binding with a ligand can be an effective strategy for achieving a biological effect [55].

After the incubation of the hTel23 G-quadruplex with increasing amounts of complex **16**, a significative destabilization of the G-quadruplex structure was detected, as shown by the reduction in both the dichroic bands at 272 and 290 nm and by the strong decrease in the T_M_ value (54 °C). A similar behavior, although to a lesser extent, was reported recently by Biver et al. [56], using a gold(I)(bis(1-(anthracen-9-ylmethyl)-3-ethylimidazol-2-ylidene) complex toward a telomeric DNA G-quadruplex. The authors attributed the destabilization of the G-quadruplex structure to non-specific outer interactions of the complex. In our case, the destabilization process was more pronounced, likely due to the greater non-selectivity of the smaller complex **16**, which shifted the folded–unfolded equilibrium toward the unfolded form during the melting experiment. Importantly, since no destabilizing effects were observed when titrating the hTel23 G-quadruplex with the non-metal ligand, it can be concluded that the detected dichroic effects were closely related to the presence of the metal. The destabilizing features observed for complex **16** are particularly interesting in light of the recent findings on the pathogenic involvement of G-quadruplexes in the development of neurogenerative diseases, such as Amyotrophic Lateral Sclerosis (ALS) and Frontotemporal Dementia (FTD) [55,57].

**Figure 6:**
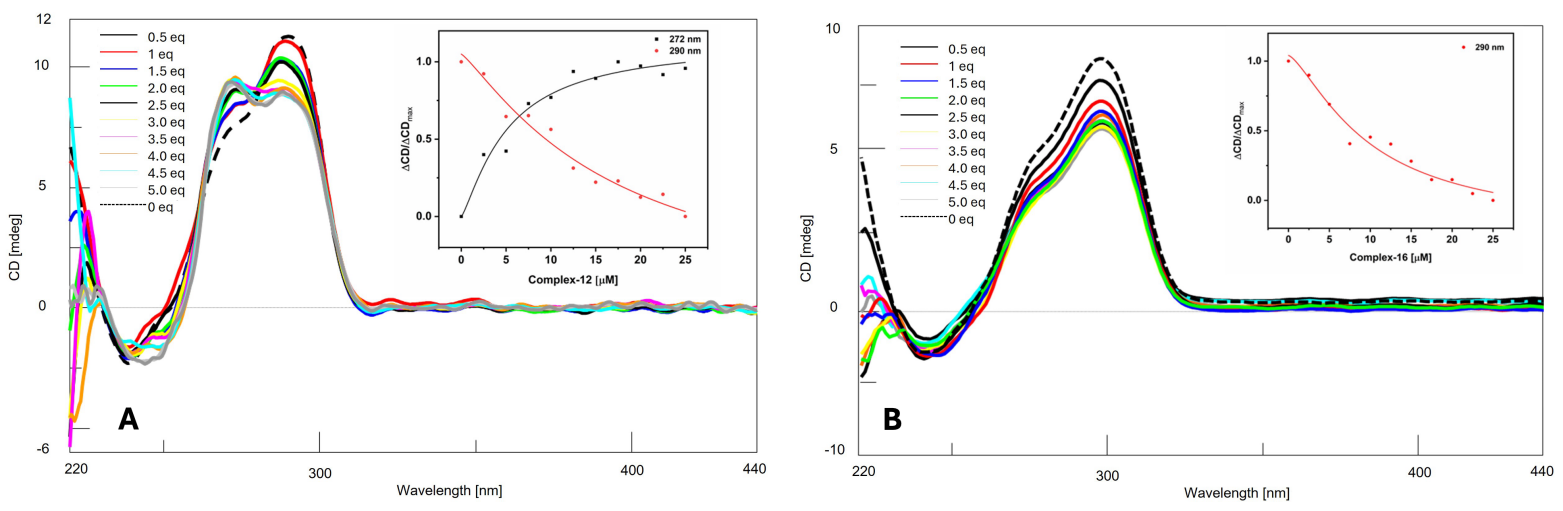
CD spectra of the hTel23 G-quadruplex in the presence of increasing molar equivalents of complexes **12** (Panel **A**) and **16** (Panel **B**). The insets show the corresponding titration curves: for complex **12**, the change in ellipticity was calculated at 272 nm (black squares) and 290 nm (red circles); for complex **16**, the change in ellipticity was calculated at 290 nm. All curves were fitted using non-linear regression (Hill fit for the CD signal at 272 nm and Hill1 fit for the CD signal at 290 nm) in OriginPro2018.

The apparent K_d_ values (Table 2) for the interaction of complexes **12** and **16** with the hTel23 G-quadruplex were derived from the CD titration plots by fitting the changes in ellipticity at 272 nm and 290 nm using the Hill equation, respectively (Figure 6) [58,59].

Complex **12** showed a relatively high affinity for the parallel conformation, with a K_d_ of 5.1 μmol/L. However, its binding to the hybrid (3 + 1) conformation was much weaker, with a K_d_ of 17 μmol/L, indicating a structural preference for the parallel form. In contrast, complex **16** predominantly bound the hybrid (3 + 1) conformation with a K_d_ of 9.7 μmol/L, showing no significant binding to the parallel form. The stronger binding affinity of complex **16** for the hybrid (3 + 1) structure highlighted its potential specificity for this conformation. These findings align with other studies on G-quadruplex binders, where K_d_ values are typically lower than 10^−6^ mol/L, depending on the structure of the ligand and the G-quadruplex topology. [60]

### 2.4. Non-Denaturing Polyacrylamide Gel Electrophoresis (PAGE)

**Figure 7:**
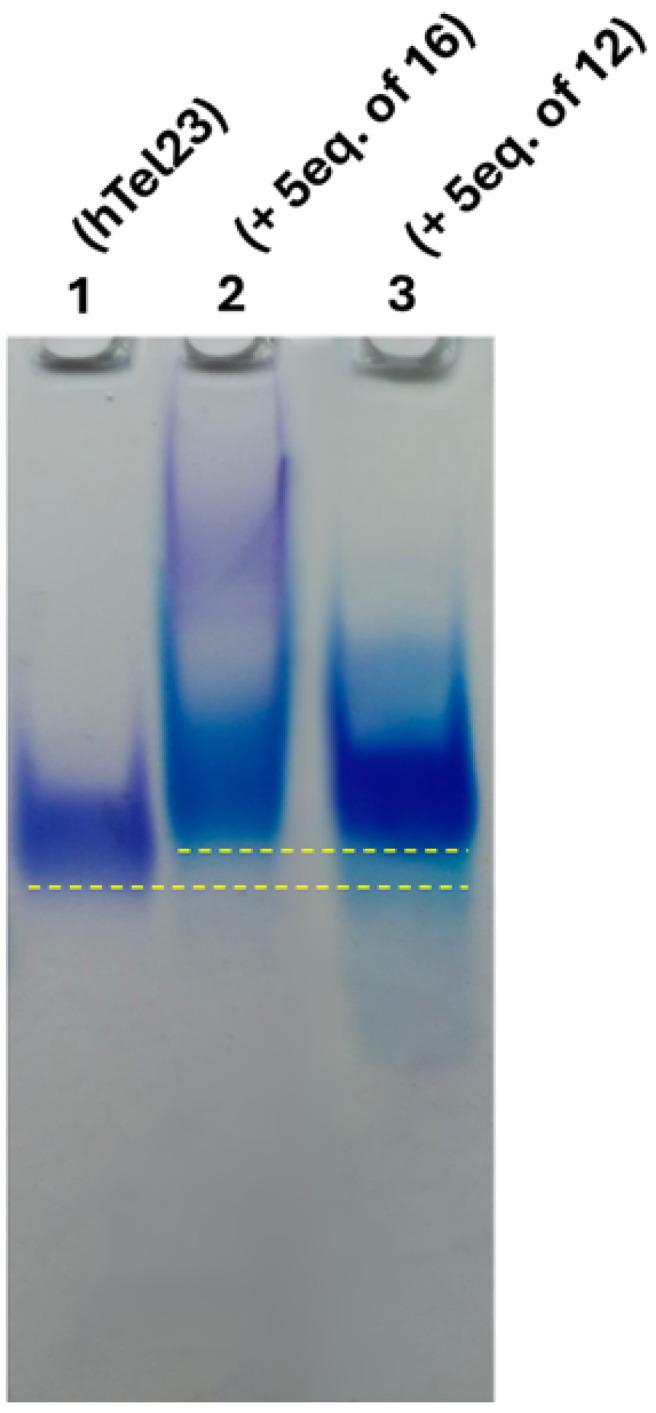
Non-denaturing PAGE prepared in TBE buffer supplemented with 30 mmol/L KCl. hTel23 G-quadruplex alone at concentration of 20 µmol/L (lane 1); hTel23 + 5 equiv. of complex **16** (lane 2); hTel23 + 5 equiv. of complex **12** (lane 3).

The non-denaturing PAGE experiment was conducted to examine the effects of the two most interesting complexes, **12** and **16**, on the conformation of the hTel23 G-quadruplex (Figure 7).

Lane 1, containing only the hTel23 G-quadruplex, showed a single, well-defined band (Figure 6), which represented the expected intramolecular hybrid (3 + 1) topology and excluded the presence of other topologies or higher-order species.

Lane 2, containing the hTel23 G-quadruplex incubated with 5 equiv. of complex **16**, displayed a prominent band along with a noticeable smearing. The faster migrating band showed an electrophoretic mobility that was slightly slower than the hTel23 G-quadruplex band in lane 1, suggesting the formation of a higher molecular weight species due to the interaction between the hTel23 G-quadruplex and complex **16**. The observed smearing further indicated that the hTel23 G-quadruplex/**16** adduct was not very stable. These results were consistent with the CD data, demonstrating the G-quadruplex-destabilizing effects induced by complex **16**.

In lane 3, where the hTel23 G-quadruplex was incubated with 5 equiv. of complex **12**, a single band was observed, displaying an electrophoretic mobility that was slightly slower than that of the unbound hTel23 G-quadruplex. This latter result suggested that the interaction between the hTel23 G-quadruplex and complex **12** led to the formation of a relatively stable complex, as indicated by the absence of smearing. All together, these data indicated that complex **12** was bound to the hTel23 G-quadruplex and formed a higher molecular weight complex.

## 3. Materials and Methods

### 3.1. General Methods

All reagents and solvents were commercially available and used without further purification. TLC analyses were performed on 0.2 mm thick F254 silica gel plates (Merck, Darmstad, Germany). TLC spots were detected under UV light (254 nm). Column chromatography was performed on silica gel-60 (Merck, 0.063–0.200 mm). The HPLC purifications were performed on a Jasco PU-4180 Plus instrument equipped with a Jasco UV-4075 Plus UV detector (Jasco Europe s.r.l., Milan, Italy) using a Nucleogel^®^ SAX 1000–8 strong anion exchange column (Macherey-Nagel, Duren, Germany) eluted with a linear gradient of 1 mol/L NaCl and 20 mmol/L NaH_2_PO_4_ aqueous solution (pH = 7.3) containing 20% (*v*/*v*) CH_3_CN in H_2_O (from 0 to 100% in 45 min., flow rate of 1.0 mL/min.). The desalinations were carried out on a Biogel-P2 column (Bio-Rad Laboratories s.r.l., Segrate, Italy). UV spectra were acquired on a Jasco V-730 spectrophotometer. CD spectra were recorded on a Jasco 1500 spectropolarimeter equipped with a Jasco PTC-348-WI temperature controller. ^1^H NMR spectra were acquired on Bruker Avance Neo 400 MHz spectrometer using DMSO-*d*_6_ as solvent. NMR chemical shifts are reported in parts per million (δ) relative to the residual solvent signals: (CH_2_D)CD_3_SO 2.54. The NMR spectra were processed using the MestReNova suite (Mestrelab Research, Santiago de Compostela, Spain, 14.3.1 version). ESI-MS spectra were acquired in positive mode on an LTQ-XL mass spectrometer equipped with an ion trap (ThermoScientific, Waltham, MA, USA).

### 3.2. Chemistry

#### 3.2.1. 2-(2-Acetamidoethyl)-2H-imidazo[1,5-a]pyridin-4-ium Chloride **19**

To a stirring solution of compound **18** (102 mg, 1.0 mmol) in EtOH (2.0 mL), paraformaldehyde (45 mg, 1.5 mmol) was added. The resulting mixture was stirred at r.t. for 4 h at which point the solution became homogeneous. Thereafter, 3.0 mol/L HCl in EtOH (0.33 mL) and 2-pyridinecarboxaldehyde (107 mg, 1.0 mmol) were added and the resulting mixture was stirred at r.t. for 16 h. Next, the solvents were removed under reduced pressure. Purification of the crude over a silica gel column (CH_2_Cl_2_/MeOH, 85:15) resulted in pure compound **19** as a colorless oil. (110 mg, yield: 48%).

^1^H NMR (400 MHz, DMSO-*d*_6_) δ 9.81 (s, 1H), 8.65 (d, *J* = 7.2 Hz, 1H), 8.27 (s, 1H), 8.16 (s, 1H), 7.88 (d, *J* = 10.4 Hz, 1H), 7.27 (ddd, *J* = 9.3, 6.6, 1.0 Hz, 1H), 7.19 (td, *J* = 6.9, 1.2 Hz, 1H), 4.56–4.51 (m, 2H), 3.57 (q, *J* = 5.8 Hz, 2H), 1.79 (s, 3H). ESI MS *m*/*z* 204, ([M^+^] calcd. for C_11_H_14_N_3_O 204).

#### 3.2.2. 2-(2-Acetamidoethyl)-2H-imidazo[1,5-a]pyridin-4-ylium Hexafluorophosphate **20**

Compound **19** (50.0 mg, 0.210 mmol) was dissolved in water (1.5 mL). The obtained solution was added dropwise to a solution of KPF_6_ (42 mg, 0.23 mmol) in water (1.5 mL). The resulting mixture was stirred at r.t. for 1 h and then extracted with AcOEt containing 5% MeOH (3 × 10 mL). The resulting mixture was stirred at r.t. for 1 h and then extracted with AcOEt containing 5% MeOH (3 × 10 mL). The combined organic layers were washed with brine, dried over Na_2_SO_4,_ and then the solvents were removed under reduced pressure. After triturating the residue with Et_2_O, pure compound **20** was recovered as a white solid (49 mg, yield: 68%).

#### 3.2.3. Bis-[2-(2-Acetamidoethyl)-2H-imidazo[1,5-a]pyridin-4-ylium]-gold(I) Bromide **16**

Ag_2_O (29 mg, 0.12 mmol) was added to a stirring solution of compound **20** (50 mg, 0.20 mmol) in CH_2_Cl_2_ containing 10% of MeOH (22 mL). The resulting mixture was stirred at r.t. for 16 h in the dark. The solution containing the complex **21** was treated with AuClS(CH_3_)_2_ (30 mg, 0.10 mmol) and LiBr (180 mg, 2.0 mmol), and stirred at r.t. for additional 5 h. The obtained gray precipitate was filtered over a pad of celite. The colorless filtrate was recovered, and the solvents were removed under reduced pressure. Purification of the crude over a silica gel column (CHCl_3_/MeOH; 95:5) resulted in pure complex **16** as a microcrystalline cream (35 mg, yield: 25%).

^1^H NMR (400 MHz, DMSO-*d*_6_) δ 8.50 (dd, *J* = 7.3, 1.2 Hz, 2H), 8.00–7.95 (m, 2H), 7.91 (s, 2H), 7.67–7.59 (m, 2H), 7.06–6.97 (m, 2H), 6.90–6.82 (m, 2H), 4.42 (t, *J* = 5.6 Hz, 4H), 3.56 (q, *J* = 5.7 Hz, 4H), 1.79 (s, 6H). ESI MS *m*/*z* 603, ([M^+^] calcd. for C_22_H_26_AuN_6_O_2_ 603).

#### 3.2.4. Preparation of the hTel23 Oligonucleotide

The hTel23 oligonucleotide d(5′-TAGGGTTAGGGTTAGGGTTAGGG-3′) was prepared by solid-phase synthesis using the β-cyanoethyl phosphoramidite chemistry on a Expedite 8909 DNA synthesizer. The standard monomers were assembled over a CPG Universal Support (35 mg, 1.4 μmol) using a 1 μmol scale protocol. The oligonucleotide was detached from the solid support and deprotected by an aqueous ammonia solution treatment at 55 °C for 12 h. The combined filtrates and washings were collected and evaporated under reduced pressure. The crude was dissolved in H_2_O, purified by HPLC, and desalted, as described in General Methods. The structure of the hTel23 sequence was confirmed by ESI MS analyses. The oligonucleotide concentration was determined spectrophotometrically at λ = 260 nm and 90 °C, using the molar extension coefficient ε = 236.5 cm^−1^ L mol^−1^, as determined using the Sigma-Aldrich OligoEvaluator^TM^ web tool (www.oligoevaluator.com, accessed on 5 September 2022).

#### 3.2.5. Preparation of the hTel23 G-Quadruplex

The lyophilized hTel23 oligonucleotide was dissolved in deionized water containing 80 mmol/L KCl and 10 mmol/L KH2PO4 (90 mmol/L K^+^ phosphate-buffered solution). The pH was adjusted at 7.3 using KOH. The oligonucleotide was annealed at a final 5 mmol/L concentration by heating the solution at 90 °C for 5 min. This was then slowly cooled to room temperature over 12 h and stored at 5 °C for 24 h before analyses. The solution was equilibrated at 25 °C for 2 h before performing the experiments.

### 3.3. CD Experiments

CD spectra were acquired in the range of 220–650 nm at 5 °C using quartz cuvettes with 0.1 cm optical path in a 90 mmol/L K^+^ phosphate-buffered solution (pH = 7.3) at the final oligonucleotide concentration of 5 μmol/L. All CD spectra were averaged over four scans recorded at 200 nm/min scanning speed, 4 s response time, and 2 nm bandwidth. The buffer baseline was subtracted from each spectrum. CD titration spectra were recorded in 90 mmol/L K^+^ phosphate-buffered solution (pH = 7.3) at 5 °C using a 5 μmol/L hTel23 G-quadruplex structure, to which increasing amounts of complexes **4**, **9**, **12**, and **16** (0.5, 1, 1.5, 2, 2.5, 3, 3.5, 4, 4.5, 5 equivalents) dissolved in DMSO were added. The max DMSO concentration used was 0.9%. The CD spectra were acquired after a 10 min incubation time. The CD melting experiments were performed by monitoring the CD value of the higher positive Cotton effect in the temperature range of 5–90 °C at the 0.5 °C/min heating rate.

### 3.4. Non-Denaturing Polyacrylamide Gel Electrophoresis (PAGE)

Non-denaturing gel electrophoresis experiments were performed using a 15% polyacrylamide gel containing 1× TBE (89 mmol/L Trizma^®^ base, 89 mmol/L borate, 2 mmol/L EDTA) and 30 mmol/L KCl, at pH 7.3. The running buffer contained 1× TBE and 30 mmol/L KCl at pH 7.3. Electrophoresis was conducted at 4 °C and 120 V for 40 min. Before loading, each sample was diluted with 1× TBE buffer containing 10% glycerol to achieve an oligonucleotide concentration of 20 μmol/L. A tracking dye was used to monitor the gel’s progress. The gel was subsequently stained with Stains-All (Merck, Darmstadt, Germany), and then it was photographed using a smartphone camera.

### 3.5. Molecular Docking Simulations

Molecular docking simulations were performed to select promising candidates as telomeric DNA G-quadruplex binders. To this aim, the X-ray structure of the complex of a human telomeric DNA with bis(1-butyl-3-methyl-imidazole-2-ylidene) Au(I) (**5**, PDB code: 6H5R, Resolution: 2.00 Å) was employed [33]. During the first step, the PDB file of the complex was submitted to the preparation phase, using the Protein Preparation Wizard tool, available in the Schrodinger suite (Schrödinger, LLC, New York, NY, USA, 2019). This tool allowed for the addition of missing hydrogen atoms, the optimization of the hydrogen bond network by reorienting the misoriented groups, the assignment of ionization states at physiological pH, and the elimination of all water molecules. Finally, after extracting the co-x, the protein was exported as a MOL2 file. The 3D structures of the selected compounds, shown in Table 1, were constructed, assigning the appropriate atom types, with the “3D builder” tool implemented in Maestro (3D Builder, Schrödinger, LLC, New York, NY, USA, 2019) and saved in aMOL2 file. Docking calculations were performed with GOLD (Genetic Optimization for Ligand Docking) [61], a docking program using a genetic algorithm (GA) for flexible docking. The hTel23 binding site was defined by selecting the centroid of one of the four co-x binding conformations (x = 0.15, y = −1.19, and z = 1.45) present in the PDB file and taking all the atoms within a radius of 10 Å. Molecular docking simulations were carried out using Gold Score (GS) as scoring function. To simulate our library, the GS parameter file was modified to properly manage the presence of dicarbene gold complexes during the simulations as described in Sciortino et al. [36], while 50 runs with a minimum of 100,000 GA operations per docking were set for the calculation. Finally, full flexibility was given to the ligands, while the receptor (tel23) was kept rigid. Such a protocol was validated by redocking the cognate ligand co-x (RMSD equal to 1.47 Å with respect to the e X-ray conformation considered). GOLD returned poses for each ligand classified according to the GS and those responsible for the best score were visually inspected.

## 4. Conclusions

Finding new molecular targets is proving to be of particular importance for cancer treatment. Among those, G-quadruplex structures are attracting significant interest from the scientific community, especially considering recent discoveries of their in vivo formation. Small molecules capable of interacting with G-quadruplex structures represent a major advancement in targeted and selective therapies, and CD spectroscopy allows for the rapid screening of potential G-quadruplex ligands. In this study, we selected a set of four NHC–gold(I) complexes based on an in silico analysis, which indicated their ability to interact with the crystallographic parallel structure of the hTel23 G-quadruplex. Since the hTel23 G-quadruplex predominantly adopts an intramolecular hybrid (3 + 1) conformation in solution, we evaluated, through CD spectroscopy, the capacity of the selected complexes to induce a conformational transition to the parallel form upon interaction with the oligonucleotide. While complex **12** promoted a shift from the hybrid (3 + 1) topology to a predominantly parallel G-quadruplex conformation, complex **16**, for which an optimized synthesis is described here, significantly destabilized the hybrid (3 + 1) structure, as further demonstrated by PAGE experiments. CD spectroscopy experiments confirmed that the observed changes in the G-quadruplex were caused by direct interaction with these complexes and not with their free ligands, highlighting the importance of the metal center in the process.

Studies are ongoing to determine the interaction mode of complex **12** with the hTel23 G-quadruplex. In addition, given the recent evidence of the pathological involvement of G-quadruplexes in neurodegenerative diseases, the interaction of complex **16** with such structures will also be investigated. In conclusion, our study aligns with the very recent discoveries in the field of G-quadruplexes, underscoring the importance of identifying ligands that could act either as destabilizers or promote transitions in the G-quadruplex structures.

## Figures and Tables

**Table 1:** Thermal effects induced by complexes **4**, **9**, **12**, and **16** (5 equiv.) added to the hTel23 G-quadruplex and measured by CD melting experiments.

Entry	λ_max_ (nm)	λ_min_ (nm)	T_M_ (°C)	ΔT_M_ (°C) *
hTel23 G4 ^#^	290	240	67	-
hTel23 G4 + **4**	290	238	65	−2
hTel23 G4 + **9**	291	244	68	+1
hTel23 G4 + **12**	272/287	250	63	−4
hTel23 G4 + **16**	290	242	54	−13

^#^ G4: G-Quadruplex. * ΔT_M_ = T [hTel23 G4 + (5 equiv. complexes **4**, **9**, **12**, and **16**)] − T [hTel23 G4].

**Table 2:** K_d_ values calculated after incubation of hTel23 G-quadruplex with complexes **12** and **16**.

	K_d_ (µmol/L)	
Entry	∆CD/∆CD_max_ Calculated at 272 nm	∆CD/∆CD_max_ Calculated at 290 nm
**12**	5.1	17
**16**	-	9.7

## Data Availability

Data are contained within the article and Supplementary Materials.

## Supplementary Materials

The following supporting information can be downloaded at https://www.mdpi.com/article/10.3390/molecules29225446/s1, Figures S1–S4: UV titration spectra of complexes **4**, **9**, **12**, and **16**.
