# Supplementary material for: A CD Study of a Structure-Based Selection of N-Heterocyclic Bis-Carbene Gold(I) Complexes as Potential Ligands of the G-Quadruplex-Forming Human Telomeric hTel23 Sequence
Source: Molecules. 2024 Nov 19;29(22):5446. doi: 10.3390/molecules29225446 (PMC11597854; doi:10.3390/molecules29225446)
Supplement: Supplementary file 1 [file molecules-29-05446-s001.zip › molecules-3291550-supplementary.pdf]

Supplementary Materials

A CD study of a structure-based selection of N-heterocyclic bis-carbene gold(I) complexes as potential ligands of the G-quadruplex forming human telomeric hTel23 sequence

Maria Marzano [1], Filippo Prencipe [2], Pietro Delre [3,4], Giuseppe Felice Mangiatordi [3], Gabriele Travagliante [5], Luisa Ronga [6], Gennaro Piccialli [4], Michele Saviano [7,8], Stefano D'Errico [4,*], Diego Tesauro [4,8,*], Giorgia Oliviero [9]

[1] CESTEV, University of Napoli Federico II, via Tommaso De Amicis 95, 80145 Napoli, Italy; maria.marzano@unina.it
[2] Department of Chemical and Pharmaceutical Sciences, University of Trieste, Via Licio Giorgieri 1, 34127 Trieste, Italy; filippo.prencipe@units.it
[3] Institute of Crystallography (IC), CNR, Via Amendola 122/O, 70126 Bari, Italy; pietro.delre@unina.it (P.D.); giuseppefelice.mangiatordi@cnr.it (G.F.M)
[4] Department of Pharmacy, University of Naples Federico II, via Domenico Montesano 49, 80131 Napoli, Italy; Pietro.delre@unina.it (P.D.); picciall@unina.it (G.P.); stefano.derrico@unina.it (S.D.); diego.tesauro@unina.it (D.T.)
[5] Department of Chemical Sciences, University of Catania, viale Andrea Doria 6, 95125 Catania, Italy
[6] Institute of Analytical and Physical Chemistry for the Environment and Materials (IPREM-UMR 5254), Université de Pau Et Des Pays de L'Adour, E2S UPPA, CNRS, 64053 Pau, France; luisa.ronga@univ-pau.fr
[7] Institute of Crystallography (IC), CNR, Via Vivaldi 43, 81100 Caserta, Italy; michele.saviano@cnr.it
[8] Interuniversity Research Centre on Bioactive Peptides (CIRPEB), University of Naples Federico II, 80134 Naples, Italy.
[9] Department of Molecular Medicine and Medical Biotechnology, University of Napoli Federico II, via Sergio Pansini 5, 80131 Napoli, Italy; golivier@unina.it

* Correspondence: stefano.derrico@unina.it (S.D.); diego.tesauro@unina.it (D.T.)

UV titration spectra of complex **4** Figure S1

UV titration spectra of complex **9** Figure S2

UV titration spectra of complex **12** Figure S3

UV titration spectra of complex **16** Figure S4

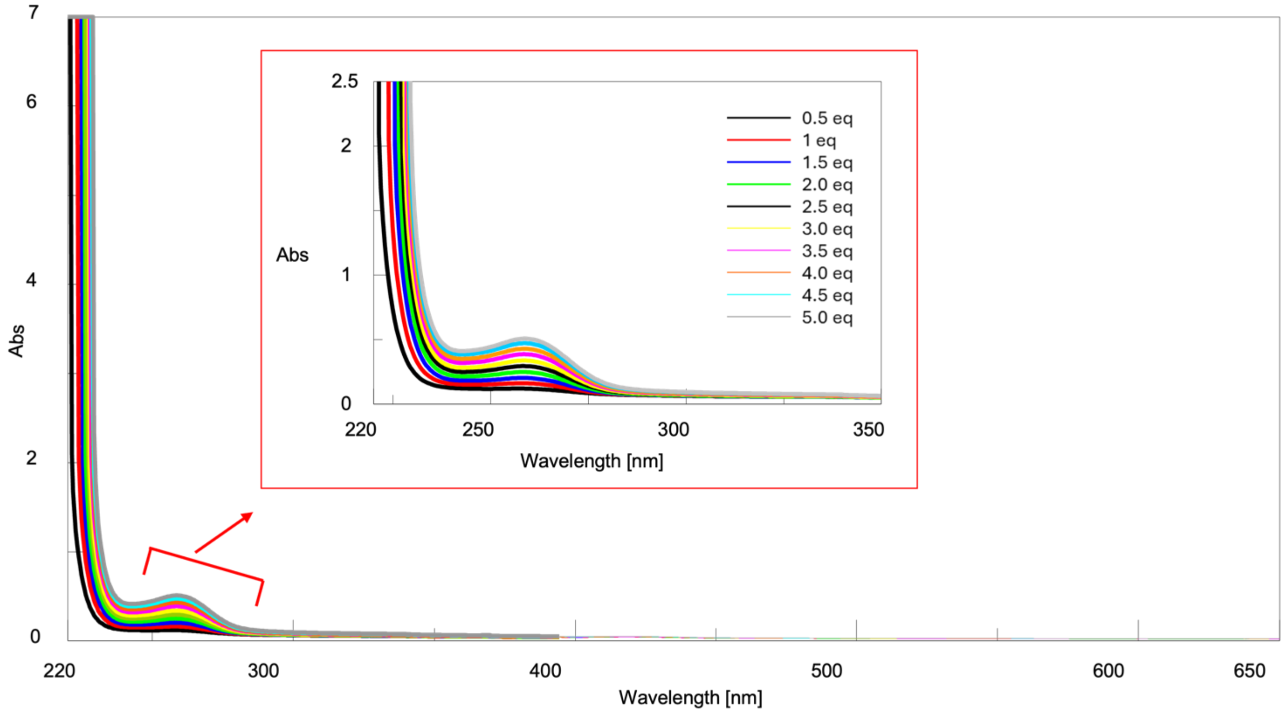

Figure S1. UV titration spectra of complex **4** in a 90 mmol/L K^+ phosphate buffered solution (pH = 7.3).

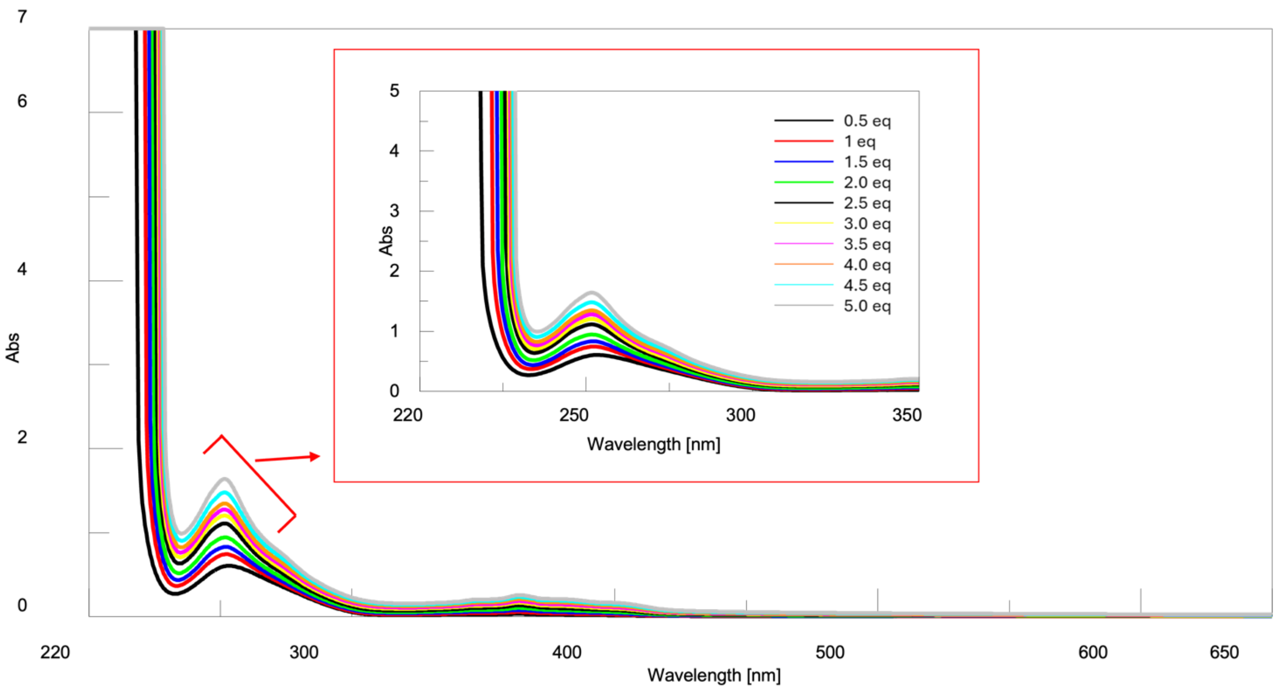

Figure S2. UV titration spectra of complex **9** in a 90 mmol/L K^+ phosphate buffered solution (pH = 7.3).

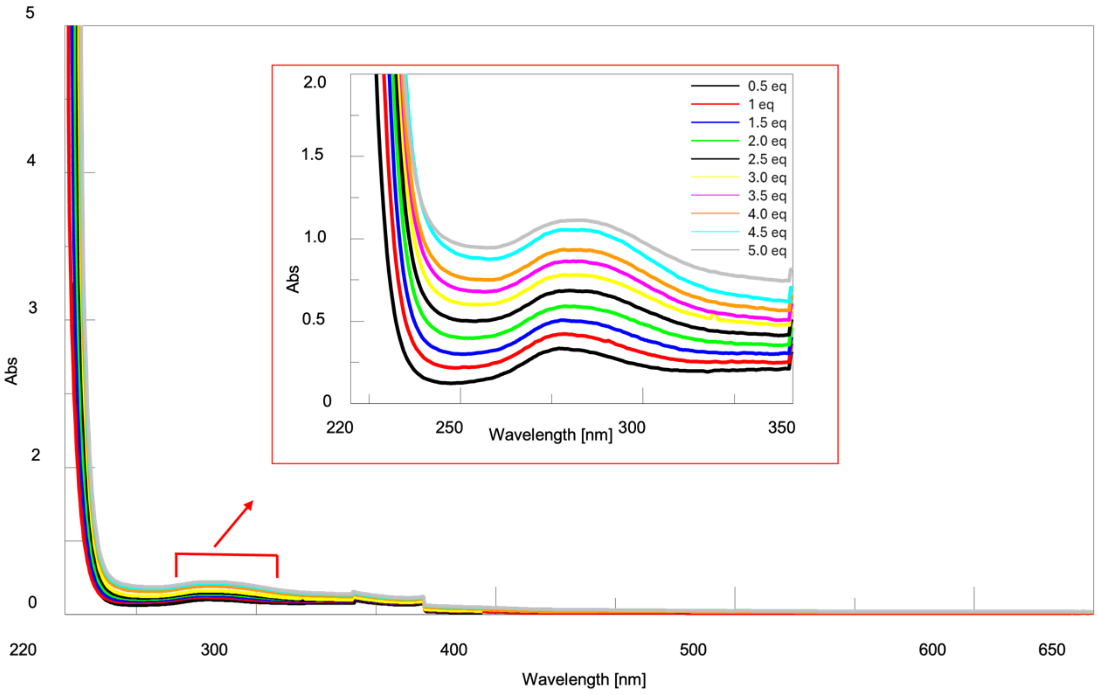

Figure S3. UV titration spectra of complex **12** in a 90 mmol/L K^+ phosphate buffered solution (pH = 7.3).

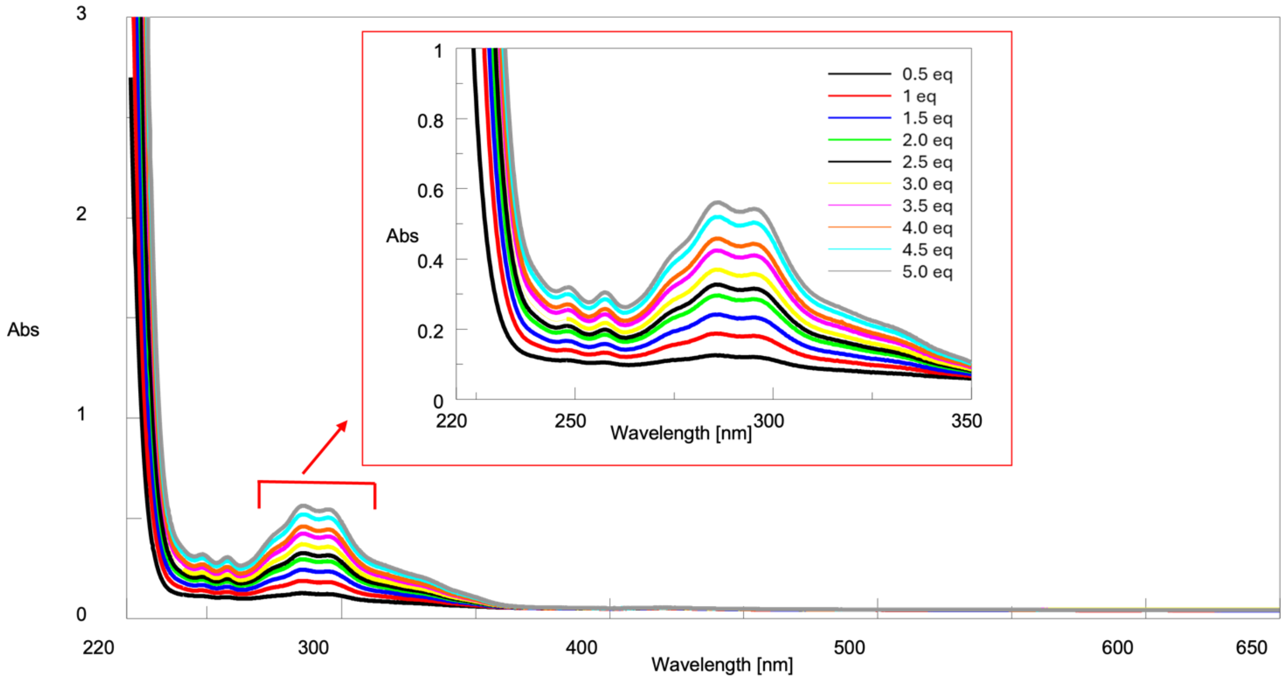

Figure S4. UV titration spectra of complex **16** in a 90 mmol/L K^+ phosphate buffered solution (pH = 7.3).
